# Double emulsion-pretreated microwell culture for the in vitro production of multicellular spheroids and their in situ analysis

**DOI:** 10.1038/s41378-021-00267-w

**Published:** 2021-05-24

**Authors:** Fuyang Qu, Shirui Zhao, Guangyao Cheng, Habibur Rahman, Qinru Xiao, Renee Wan Yi Chan, Yi-Ping Ho

**Affiliations:** 1 Department of Biomedical Engineering, Faculty of Engineering, The Chinese University of Hong Kong, Shatin, New Territories, Hong Kong SAR, China; 2 CUHK-UMCU Joint Research Laboratory of Respiratory Virus and Immunobiology, The Chinese University of Hong Kong, Shatin, New Territories, Hong Kong SAR, China; 3 Department of Paediatrics, Faculty of Medicine, The Chinese University of Hong Kong, Shatin, New Territories, Hong Kong SAR, China; 4 Laboratory for Paediatric Respiratory Research, Li Ka Shing Institute of Health Sciences, Faculty of Medicine, The Chinese University of Hong Kong, Hong Kong SAR, China; 5 Hong Kong Hub of Paediatric Excellence, The Chinese University of Hong Kong, Hong Kong SAR, China; 6 Hong Kong Branch of CAS Center for Excellence in Animal Evolution and Genetics, The Chinese University of Hong Kong, Shatin, New Territories, Hong Kong SAR, China; 7 The Ministry of Education Key Laboratory of Regeneration Medicine, Shatin, New Territories, Hong Kong SAR, China; 8 Centre for Novel Biomaterials, The Chinese University of Hong Kong, Shatin, New Territories, Hong Kong SAR, China

**Keywords:** Nanoscience and technology, Nanobiotechnology, Microfluidics

## Abstract

Multicellular spheroids have served as a promising preclinical model for drug efficacy testing and disease modeling. Many microfluidic technologies, including those based on water–oil–water double emulsions, have been introduced for the production of spheroids. However, sustained culture and the in situ characterization of the generated spheroids are currently unavailable for the double emulsion-based spheroid model. This study presents a streamlined workflow, termed the double emulsion-pretreated microwell culture (DEPMiC), incorporating the features of (1) effective initiation of uniform-sized multicellular spheroids by the pretreatment of double emulsions produced by microfluidics without the requirement of biomaterial scaffolds; (2) sustained maintenance and culture of the produced spheroids with facile removal of the oil confinement; and (3) in situ characterization of individual spheroids localized in microwells by a built-in analytical station. Characterized by microscopic observations and Raman spectroscopy, the DEPMiC cultivated spheroids accumulated elevated lipid ordering on the apical membrane, similar to that observed in their Matrigel counterparts. Made possible by the proposed technological advancement, this study subsequently examined the drug responses of these in vitro-generated multicellular spheroids. The developed DEPMiC platform is expected to generate health benefits in personalized cancer treatment by offering a pre-animal tool to dissect heterogeneity from individual tumor spheroids.

## Introduction

The success rates of cancer clinical trials remain relatively low despite substantial progress made in the past decades^1^. Interwoven cell–cell interactions may sway the prediction of how a population of cells will respond to a given drug treatment^2^. Further, cancer is notoriously complex and is characterized by profound heterogeneity between patients and even between malignant cells within a single tumor^3,4^. In response to this complexity, most efforts have focused on cancer genomics and analyzing the mutation profiles of patient tumors; e.g., the activating V600E mutation in the BRAF gene that is frequently observed in primary melanomas and papillary thyroid carcinomas^5^. However, the autonomous sources of cell heterogeneity (i.e., genetics, epigenetics, and other factors not determined by the microenvironment) illustrate only part of the complexity, as the highly variable tumor microenvironment may also contribute to cell variability and in turn significantly affect therapeutic outcomes^4,6^. Thus, limitations remain, because studies performed to date rely largely on ensemble investigations in two-dimensional culture dishes, hindering the assessment of the characteristics of cell–cell interactions as well as extracellular matrix (ECM) architectures^7^.

Efforts to overcome these obstacles have led to the development of in vivo-mimicking three-dimensional (3D) culture models such as multicellular tumor spheroids^8^. Mounting evidence has demonstrated that ex vivo cultivated tumor spheroids behave very differently from monolayer cultured cells and reflect many features of solid tumors^8–10^. For example, MMP2, a biomarker involved in mesothelioma pathogenesis, has been observed to be highly expressed in mesothelioma spheroids produced by 3D culture of the human mesothelioma cell line NCI-H226 in a matrix-free format^11^. The 3D construct of spheroids resembles solid tumors by the presence of a central necrotic and hypoxic core, which plays an important role in initiating the signaling pathway and transcriptional alterations to promote cancer cell survival, as well as the generation of cancer stem cells, two factors that are considered to be closely associated with drug resistance and tumor relapse^12^.

Currently available strategies to culture tumor spheroids include culture in both scaffolds and a scaffold-free manner (such as in suspension, in a spinner flask, in hanging drops and on nonadherent surfaces)^13^. For scaffold-based cultivation, Matrigel, a soluble basement membrane extract secreted by mouse sarcoma cells, is perhaps the most widely employed matrix. The rich biochemical cues such as collagen IV, laminin-111, entactin, heparin sulfate proteoglycan, and several vital growth factors, as well as the spider-web-like matrices provided by Matrigel, have shown high analogy with the in vivo tumor microenvironment^14^. Therefore, the multicellular spheroids cultivated in Matrigel demonstrate complex cellular behaviors that are otherwise difficult to obtain through other culture approaches. Nevertheless, the ability of Matrigel to encourage in vivo-like cellular behavior originates from the complex and heterogeneous compositions of Matrigel, which consequently renders batch-to-batch variation^15^. Of note, the role of the laminin-rich artificial ECM in the expression profiling of 3D tumor culture remains ambiguous^11^. Practically speaking, these animal-derived scaffolds are often costly, in particular when large-scale drug screening is considered. Culturing tumor spheroids in a scaffold-free microenvironment relies on the ability of cancer cells to spontaneously cluster with each other through the secreted ECM^16^. Scaffold-free approaches are relatively accessible and easy to operate; however, the commonly recognized drawbacks are the uncontrolled size and configuration of the spheroids, as well as the potential shear-induced cell damage, especially when spinner flasks are engaged^17^. Among the available approaches, culture in hanging drops has provided improved controllability of the spheroid size^9,18^; however, the spheroid size hinges heavily on the smallest dispensable volume from a handheld pipette. Furthermore, the drop dispensing process is labor intensive. Although recent developments, such as high-throughput droplet printing, have shown accelerated spheroid production, the equipment may not be very affordable. Culture on nonadhesive surfaces, such as agar^19^ and poly-2-hydroxyethyl methacrylate^20,21^, can produce large numbers of spheroids in a high-throughput format; however, the spheroid size is normally variable and thus a mixture of different morphologies of spheroids and unclustered cells. Furthermore, given the scenario of having the spheroids cultured in a culture flask or culture well, disturbance to the spheroids becomes an unavoidable consequence during the medium exchange step. Taken together, the tumor spheroid model holds great promise as an effective preclinical model for drug screening and precision medicine; however, the model has not reached its full potential in cancer research, mainly due to lingering technical challenges in controlled spheroid cultivation and maintenance.

To this end, micro/nanofabrication techniques, including microchambers^22^, microwells^23,24^, and microdroplets^25,26^, have emerged as potential solutions by allowing 3D culture over an extended period, as well as enabling cocultures and the signaling gradient in a spatially controlled manner^27^. Droplet platforms^28–30^ offer many advantages for spheroid formation, such as controlled droplet size (and therefore control over spheroid size), very large production rate (normally in the range of kHz), and the possibility of on-chip analysis. We previously demonstrated successful encapsulation and formation of tumor spheroids in water–oil–water double emulsions (DEs)^31^. The initiation of intestinal Caco-2 (colon colorectal) spheroids was promoted and observed 2 days after culture in a scaffold-free format inside the DEs. Further examination showed that the Caco-2 spheroids cultivated in the DEs evoked their polarity and developed a crypt-villus structure that was similar to that observed during the first 2 days of intestinal organoid initiation. The self-assembly of multicellular spheroids without any involvement from a scaffold is presumably due to confinement in the DEs that prevent the spreading and migration of cells, similar to the observed organoid generation induced by substrate nanotopography^32^. However, constrained nutrient and gas transport in DEs has limited an extended investigation of the produced spheroids.

On the other hand, recent studies have recognized the necessity of the on-chip and addressable analysis of generated spheroids. Sabhachandani et al.^33^ integrated a docking array with the generation module of water-in-oil single emulsions for long-term spheroid cultivation and functional assessment. The MCF7 spheroids were maintained for 2 weeks and their response to drug treatment was evaluated on the chip. Acknowledging the drawback of the compartmentalization of single emulsions, McMillian et al.^34^ developed a storage array to trap single emulsions containing spheroids for subsequent coalescence, thereby enabling the injection of fresh media and drug treatment. A human glioma cell line was cultivated into spheroids and the response of these cells to X-ray radiation was evaluated by their size and a viability assay. Sart et al.^35^ entrapped single emulsions containing cell-liquid agarose onto a culture well array. Fluorocarbon oil was washed away to ensure the long-term survival of the organoids produced by human mesenchymal stromal cells for subsequent investigations. However, an on-chip analysis platform is currently unavailable for DE-produced spheroids.

As a continuous effort to prolong the investigation and expand the capacity of the in situ analysis of DE-produced tumor spheroids, this study presents a streamlined platform by combining DE-based spheroid initiation and microwell culture, henceforth termed DE-pretreated microwell culture (DEPMiC). Calu-3 (*Homo sapiens* lung adenocarcinoma) cells were selected as a model for their morphologically obvious cyst structure as an indication of differentiated tumor spheroids. Aligned with our previous observations, Calu-3 spheroids exhibited cyst structures after 1 day of “pretreatment” in DEs. An emulsion trapper was developed to entrap the DEs individually in geometrically well-fitted microwells. After breaking the oil layers of the DEs, the released spheroids proliferated and gradually exhibited a circular spheroid morphology, and were cultured with existing 3D culture approaches. Further investigations by Raman spectroscopy demonstrated that the Matrigel-produced spheroids and those cultured by the DEPMiC method, both present ordered lipid structures in the apical membrane as opposed to the basal membrane. We also custom-built a detection system to evaluate the drug responses from individual spheroids in an in situ manner, where the half maximal inhibitory concentration (IC50) was observed to be comparable to the drug sensitivity measured from tumor spheroids produced with patient-derived cancer-associated fibroblasts.

## Results and discussion

### Production and localization of DEs and spheroids

**Fig. 1:**
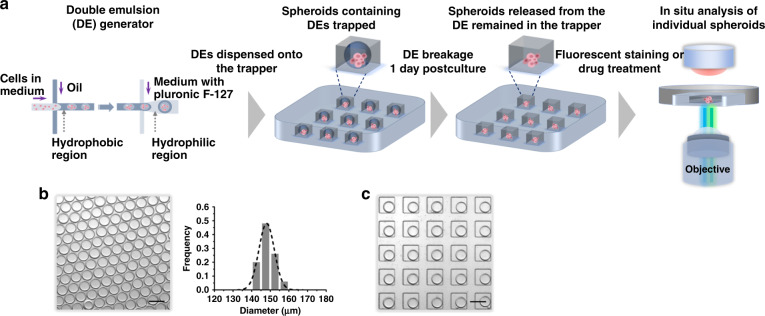
Double emulsion-pretreated microwell culture (DEPMiC). **a** The workflow of the DEPMiC. Culture medium containing cells, oil-surfactant mixture, and culture medium containing Pluronic F-127 were introduced to the emulsion generator. The generated double emulsions (DEs) are then transferred onto the emulsion trapper, where the geometry of the microwells was tailored for the produced DEs. The spheroids were observed to be unperturbed upon subsequent treatment with the DEs, such as oil layer removal, fluorescent staining, and drug treatment. The in situ analysis of individual spheroids is made possible by a customized fluorescence measurement platform. **b** A representative microscopic image (left) and size distribution of the produced DEs (right) demonstrate that the produced DEs are highly uniform with a diameter of ~150 μm. The dashed line on the histogram represents a Gaussian fit of the raw data. **c** A representative microscopic image showing that the emulsion trapper treated with BSA is capable of localizing the produced DEs. Scale bars: 200 μm.

Calu-3 cells were introduced as a model to demonstrate the workflow of the proposed DEPMiC system (Fig. 1a). Two separate microfluidic chips, a DE generator and an emulsion/spheroid trapper, were prepared to generate and localize the DEs, respectively. Culture medium containing Calu-3 cells, an oil-surfactant mixture and culture medium with Pluronic F-127 were introduced into the DE generator from the inner aqueous inlet, oil inlet, and outer aqueous inlet, respectively. Water-in-oil single emulsions were produced at the first cross-junction, whereas the water-in-oil-in-water DEs were emulsified at the second cross-junction, a hydrophilic region grafted with polyacrylic acid. The generated DEs were highly monodisperse (diameter of 150 ± 4.2 μm, shell thickness 11.9 ± 1.7 µm), as shown in Fig. 1b. These cell-containing DEs were subsequently transferred to an emulsion/spheroid trapper containing microwells (10 × 10 matrix = 100 microwells, L 200 μm × W 200 μm × H 110 μm), which were employed to streamline the production and localization of DEs for in situ analysis. Although the geometry of microwells were designed to fit with the size of the DEs, the hydrophobic methyl groups on the side chain of polydimethylsiloxane (PDMS) were attracted to the fluorocarbon chain situated in the oil layers of the DEs. As a result of minimized surface free energy^36^, fluorocarbon oil was anticipated to spread over the PDMS surface, rendering disruption of the trapped DEs. Bovine serum albumin (BSA) coating of the PDMS surface was therefore introduced to resolve this technical problem. As observed in Supplementary Fig. S1a, some DEs were broken when the PDMS surface was not treated with BSA. After surface treatment with BSA, all DEs remained intact, as shown in Fig. 1c and Supplementary Fig. S1b. For each experiment, 5 µL of sample containing DEs produced by the droplet generator was placed on top of the emulsion trapper. Considering the closely packed DEs, the corresponding number of DEs was calculated to be ~3000 based on the collected volume. Given the excess DEs vs. the number of wells (~30 times excess), the occupancy of the microwells was determined to be close to 100%. For sustained culture of the spheroids, the oil layers were then removed by adding the emulsion-releasing agent onto the trapper. The released spheroids were observed to be soundly retained in the microwells throughout the whole process from emulsion breaking and fluorescent staining to drug treatment. The spheroids entrapped in the microwells were then analyzed in situ with a custom-built detection system.

### Morphology of the spheroids cultured in the DEs

**Fig. 2:**
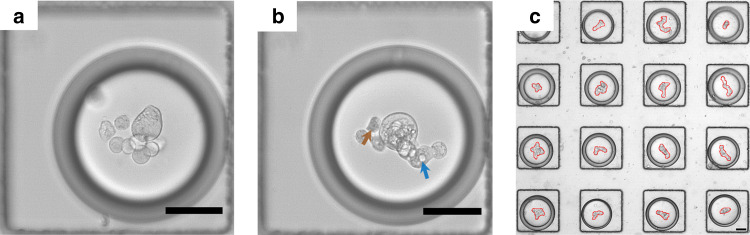
Pretreatment of Calu-3 cells in the double emulsions for 24 h. **a** DEs loaded with cells immediately after the DE generator. **b** In situ observation of the aggregation of cells 1 day post culture. A cyst-like structure comprising the features of vacuolar apical compartments, as indicated by a blue arrow, was observed in most of the DE-pretreated spheroids. Some unhealthy cells were observed dislocated from the cluster, marked by the brown arrow. **c** An overview of spheroid-containing DEs in the trapper, with the contour of each spheroid outlined in red. Scale bars: 50 μm.

As shown in Fig. 2a, Calu-3 cells loaded into a DE typically appeared to accumulate in the center of a DE, presumably owing to the curved surface surrounding the DE. To facilitate the secretion of ECM by the crowding effect^37^, Ficoll 400 and Ficoll 70 molecules were included in the cell culture medium. Similar to previous observations, cells spontaneously aggregated into spheroids as soon as they were cultured inside the DEs for 1 day (Fig. 2b). At 1 day post-DE encapsulation, cells within each spheroid appeared mostly morphologically connected (Fig. 2c); however, it is of note that a few cells were occasionally observed to be dislocated from the cluster (arrowed in brown, Fig. 2b), which may be ascribed to their unhealthy state, as indicated by the rough edges. It is noteworthy that the spheroids in the DEs were observed to have nonuniform sizes, mainly from the variation of cell number (ranging from 3 to 11) in each DE due to the Poisson distribution. More importantly, small cyst-like structures, presumably containing the vacuolar apical compartment^38^, were observed (arrowed in blue, Fig. 2b) in most of the DE-pretreated cell spheroids (Fig. 2c). Similar cyst structures were also observed in the early stage epithelial organization in vivo^39^ as well as for the spheroids cultured in Matrigel (Supplementary Fig. S3). Different from the Matrigel-cultured counterpart, the spheroids cultured in the DEs elicited irregular contours (outlined in red, Fig. 2c), which prompted us to perform subsequent characterizations.

### Cell viability in the DEs

**Fig. 3:**
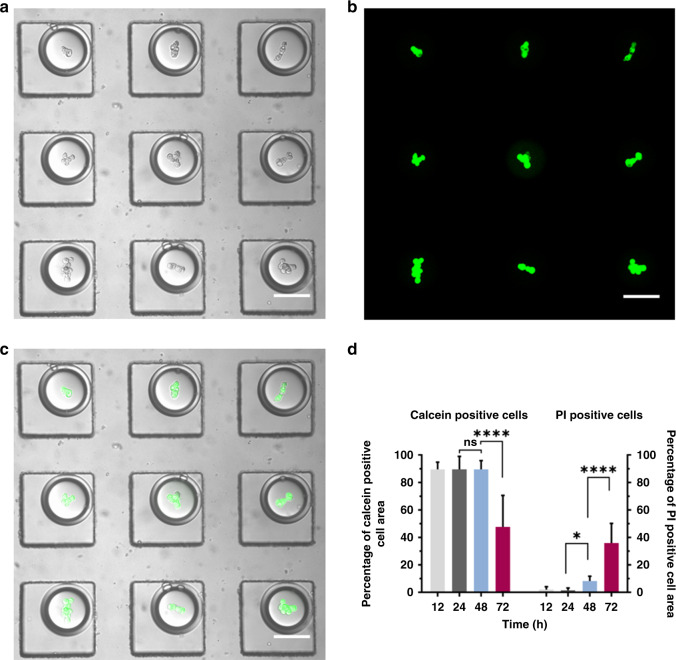
Viability of the cells cultivated in the DEs. **a** A representative bright-field image showing the spheroids 1 day post culture in the DEs. **b** The merged image of calcein- and PI-stained spheroids in **a**. **c** The merged image of **a** and **b**. Scale bars: 100 μm. **d** Percentage of calcein-positive and PI-positive cell areas were plotted at 12, 24, 48, and 72 h post culture in the DEs. Data are presented as the mean ± SD. Ordinary one-way ANOVA was used for the statistical analysis (ns no significance; *P < 0.05; ****P < 0.0001; *n* = 10).

Although the DE-pretreated culture has shown promise for spheroid initiation, it is of concern that the oil layer may retard the mass transport of nutrients and waste, particularly when long-term cultivation is considered. Made possible by the designed emulsion trapper (Fig. 3a), we tested the viability of the cells cultivated inside the DEs with calcein-acetoxymethyl (AM) and propidium iodide (PI) at different time points. As shown in Fig. 3b, c, Calu-3 cells cultured in DEs for 1 day appeared fairly healthy, as reported by the intense green fluorescence from calcein and negligible red fluorescence from PI. Serving as a quantitative understanding of cell viability, Fig. 3d was plotted as the percentage of calcein- and PI-positive cell areas by quantifying the areas stained by calcein and PI as opposed to the total cell projection area. At 48 h post culture, the PI (red)-positive area was elevated, suggesting that the cells started to suffer from apoptosis, although the calcein (green)-positive area remained minimally altered compared with the time points of 12 and 24 h post culture. On Day 3, perforation of the cell membranes was further aggravated, as indicated by the significantly increased PI-positive area, which was accompanied by a reduction in the calcein-positive area. Therefore, a combinatorial analysis of calcein and PI staining was shown to be necessary to avoid potential bias by investigating fluorescence staining solely from calcein or PI. Second, the PI-positive signal suggests that the cells cultivated in the DEs likely entered apoptosis or necrosis at 48 h post culture.

### Growth dynamics of DEPMiC-produced Calu-3 spheroids observed in situ

**Fig. 4:**
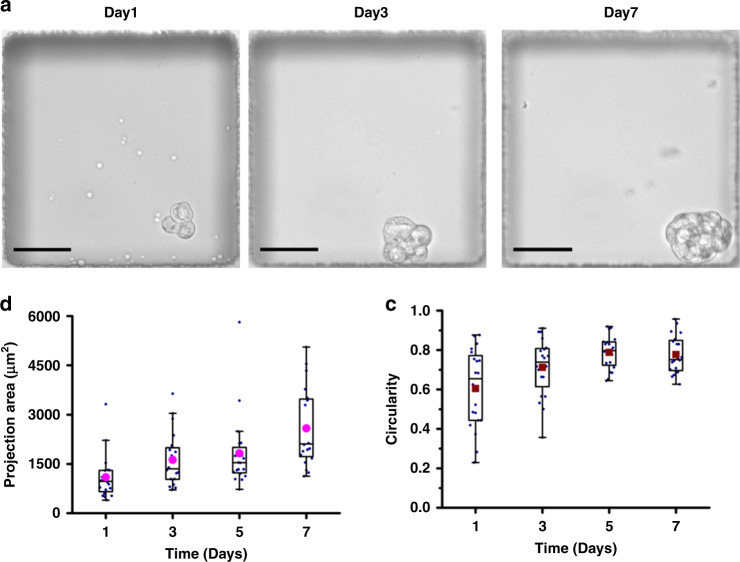
Growth dynamics of Calu-3 spheroids cultivated by DEPMiC. **a** In situ observation of the same spheroid at 1, 3, and 7 days after release from the DEs. **b** The projection area of cell spheroids within a week measured every other day. The closed purple circles, boxed region, and whiskers are plotted as the mean, interquartile range of 25%‒75%, and the nonoutlier range, respectively (*n* = 20). **c** The circularity of spheroids calculated based on the equation defined in the “Methods” section. The closed crimson squares, boxed region, and whiskers are plotted as the mean, interquartile range of 25%‒75%, and nonoutlier range, respectively (*n* = 20). Scale bars: 50 μm.

Drawing on the morphologies and viability of the cells cultivated in the DEs, prolonged confinement in DEs may potentially hinder the growth of the spheroids and lead to potential cell death. Therefore, subsequent investigations were conducted by releasing the cell spheroids “pretreated” in the DEs at 24 h post culture onto a PDMS-based microwell. The spheroids were released from the DEs by adding the DE-releasing agent nonafluoro-tert-butyl alcohol (0.2% v/v, 20 min of incubation). Prior to further assessment of the released spheroids, the DE-releasing agent was verified to have negligible cytotoxicity at a concentration of up to 0.5% (v/v), 2.5× higher than the employed concentration for emulsion breakage (Supplementary Fig. S2). As the DEs were entrapped in the microwells individually, the released spheroids were retained in the same microwells, therefore allowing an assessment of the growth of the individual spheroids, as shown in Fig. 4a. As observed from Fig. 4b, the size of the spheroids increased over a 7-day culture period, as illustrated by the increasing projection area of cell spheroids. In addition, the circularity calculated from the projected boundary of spheroids approached a plateau with a value of ~0.8 after 7 days of culture (Fig. 4c), suggesting that the cell spheroids were inclined to assemble into a spherical structure, as observed in other 3D culture methods.

### Characterization of lipid ordering by Raman spectral analysis

**Fig. 5:**
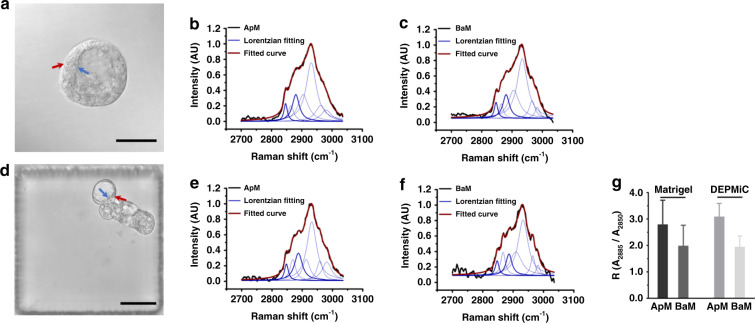
Polarity of the spheroids characterized by Raman spectroscopy. **a** A representative bright-field image taken from a spheroid cultured in Matrigel, where the apical and basal membranes are indicated by blue and red arrows, respectively. Normalized Raman spectra acquired from spheroids cultured in Matrigel were plotted in **b** for the apical membrane (ApM) and in **c** for the basal membrane (BaM). **d** A representative bright-field image taken from a spheroid cultured by DEPMiC, where the apical and basal membranes are indicated by blue and red arrows, respectively. Normalized Raman spectra acquired from the spheroids cultured by DEPMiC were plotted in **e** for the apical membrane (ApM) and in **f** for the basal membrane (BaM). For the Raman spectra, the measured raw Raman signal and the fitted line are plotted in black and crimson, respectively. The seven Lorentzian fitting curves are shown in blue, where the two peaks at 2850 cm^−1^ and 2885 cm^−1^ are bolded. **g** The ratios (*R*) between the areas under the curve for the peaks at 2850 cm^−1^ and 2885 cm^−1^, i.e., *R* = *A*_2885_/*A*_2850_, were plotted for the apical membrane (ApM) and basal membrane (BaM) measured from spheroids cultured in Matrigel and by DEPMiC. Data are presented as the mean ± SD. Scale bars: 50 μm.

Although the observed cyst structures in Fig. 2b, c may serve as a morphological indication of the organotypic feature exhibited by the DE-pretreated spheroids, it remains unclear whether the obtained spheroids may recapitulate the architecture and function of epithelial tissues, such as the manifestation of apical–basal polarity. The Calu-3 spheroids grown in Matrigel were employed as a comparison for the subsequent investigations. Similar to previous reports, Calu-3 cells cultured in Matrigel formed a solitary central lumen-containing spherical structure, as shown in Fig. 5a. As depicted by the immunostaining in Supplementary Fig. S4, the expression of an apical domain marker (ZO-1, one of the junctional complexes, located on the lateral plasma and near the apical membrane) and a basal domain marker (β1-integrin, expressed on the basal membrane) indicated that the spheroids established positive polarity^32^. On the other hand, immunostaining of the DEPMiC-produced spheroids did not show obvious ZO-1 and β1-integrin signals around the cyst structures (Supplementary Fig. S5), suggesting that ZO-1 and β1-integrin may not be expressed distinctly in the apical–basal domain of DEPMiC-produced spheroids. Immunostaining suggested that the DEPMiC-produced spheroids may manifest random polarity, presumably due to the absence of external cues, such as the ECM, to initiate polarization. However, the phenotype of polarized epithelial cells exhibits not only the various proteins expressed on apical and basal membranes but also the distinct lipid distribution on the membranes. Previously documented evidence has suggested that sphingolipids and cholesterol around the apical membrane may assemble into a liquid-ordered architecture, a higher-order structure not observed at the basal membrane^40,41^.

Recently, Raman spectroscopy has emerged as a label-free technique to profile different degrees of lipid ordering^42^. Raman spectral analysis was therefore employed to extract the lipid-ordering information intrinsically. The laser was focused on the apical and basal membranes, as indicated by the blue and red arrows in Fig. 5a, d for the spheroids cultivated by Matrigel and DEPMiC, respectively. The Raman spectra measured from the spheroids cultured by Matrigel (Fig. 5b, c) and DEPMiC (Fig. 5e, f) were processed by peak fitting. Seven Lorentzian fitted symmetric curves were extracted from each Raman spectrum, in which the peaks at 2850 cm^−1^ and 2885 cm^−1^ (two bolded blue curves) represent the symmetric and asymmetric CH_2_ stretching vibrations, respectively. In ordered lipid assemblies, the Fermi resonance has been observed to be enhanced for asymmetric CH_2_, whereas the symmetric CH_2_ stretching vibration remains unaffected^43,44^. The ratio between the areas under the curve of the two Raman shifts (*R* = *A*_2885_/*A*_2850_) was therefore employed as the degree of lipid ordering, as defined in previous findings^42–44^. Figure 5g depicts the *R*-values obtained from the apical and basal membranes of spheroids cultured by the two different 3D culture methods, namely Matrigel and DEPMiC. As expected, the ratio *R* measured from the apical membrane (*R*_ApM_) was larger than that from the basal membranes (*R*_BaM_) for both culture conditions, suggesting that ordered lipids were similarly observed at the apical membrane under both conditions. As a quantitative indication of the difference in lipid ordering between the apical and basal membranes, *R*_o_ was defined as the ratio of *R*_ApM_ to *R*_BaM_, i.e., *R*_o_ = *R*_ApM_/*R*_BaM_^42^. The *R*_o_ values acquired from the Matrigel- and DEPMiC-based cultures were 1.41 and 1.59, respectively. Although the immunostaining images of the DEPMiC-cultivated spheroids were not identical to those obtained from their Matrigel counterparts, the Raman analysis indicated that the observed difference in lipid ordering between the apical and basal membranes was similar for both cases. However, further investigations of the DEPMiC-produced spheroids are necessary to explain why the elevated lipid-ordering structure is established on the apical membrane, while the ZO-1 and β1-integrin proteins are not distinctly expressed in the apical–basal domain.

### In situ measured drug response from the DEPMiC-cultivated spheroids

**Fig. 6:**
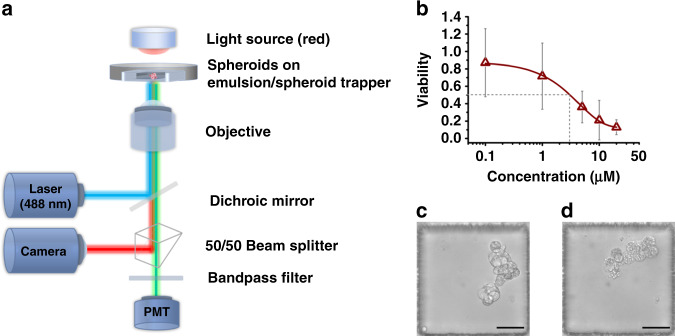
Customized analytical platform for the in situ analysis of DEPMiC-cultivated spheroids. **a** Schematic diagram of the analytical platform capable of simultaneously acquiring bright-field images and fluorescence signals. **b** The viability measured from individual spheroids under treatment with vinorelbine at titrated concentrations, where the IC50 value was determined to be ~3 μM, as marked by the gray dashed lines. **c** Morphological observations of identical spheroids before vinorelbine treatment and **d** after treatment with vinorelbine (20 μM) for 3 days. Data are plotted as the mean ± SD (*n* = 3 independent experiments). Scale bars: 50 μm.

To demonstrate the feasibility of the proposed workflow of DEPMiC as a streamlined platform for the in situ characterization of spheroids, drug efficacy testing was subsequently performed. As shown in Fig. 6a, a customized detection system was constructed for bright-field imaging and measurement of the averaged fluorescence signal from individual spheroids. Vinorelbine, a drug commonly employed to treat non-small cell lung cancer, was incubated with the spheroids cultivated in the trapper. Analysis of these DEPMiC-cultivated spheroids was conducted in situ in the microwells. Bright-field images and fluorescence signals from calcein were acquired simultaneously from each microwell. The responses of each spheroid under vinorelbine treatment were plotted as cell viability vs. the titrated concentration of vinorelbine, as shown in Fig. 6b, where the IC50 value was identified to be ~3 μM. The estimated IC50 corroborated well with the drug sensitivity of ~2.5 μM, which was found in a study where tumor cells from a group of non-small-cell lung cancer (NSCLC) patient-derived xenograft lines were cultured in a low-stiffness laminin-rich ECM supplemented with patient-derived cancer-associated fibroblasts^45^. Notably, appreciable variation was observed at low concentrations of vinorelbine. Considering that the data were acquired from individual spheroids, the observation variation may be attributed to the intrinsic heterogeneity of the individual spheroids. More importantly, the DEPMiC-produced spheroids may exhibit random polarity, as manifested by the immunostaining results, whereas the alignment of microtubules was observed parallel along the apical–basal axis for polarized cells^46^. Randomly polarized cells in spheroids may result in varied distribution of the plus ends of the microtubules, where tubulin dimers are added or removed to change microtubule length^47^. In addition, the vinorelbine employed in this test may also inhibit the assembly of microtubules^48^. Considering that the drugs may penetrate cells without directional preference, the randomly polarized cells might display various responses to the drug and therefore lead to the variation observed, particularly at low drug concentrations.

Considering that the Calu-3 cell line is established from NSCLC, the developed workflow of DEPMiC may serve as a good candidate for drug screening and/or in situ analysis of spheroids cultivated in a 3D format. Furthermore, real-time investigation of individual spheroids is also possible, as demonstrated by the morphological changes of the identical spheroid shown in Fig. 6c, d before and 3 days after vinorelbine treatment.

## Conclusions

This study presents a streamlined platform for spheroid formation, cultivation, and in situ characterization. Microfluidics-generated DEs have been employed as a pretreatment to encourage spheroid initiation, whereas microwell culture made the localization of spheroids possible for subsequent in situ analysis. Without artificial scaffolds, Calu-3 cells cultivated by the DEPMiC method exhibited cyst structures after 1 day of pretreatment in DEs and the circular morphology resembled the phenomena observed in their Matrigel counterparts. Notably, the DEPMiC-produced spheroids do not distinctly express the ZO-1 and β1-integrin proteins around the cyst structures, whereas the ZO-1 and β1-integrin were typically observed in the Matrigel counterparts. However, investigations by Raman spectroscopy demonstrated that the DEPMiC-produced spheroids present ordered lipid structures in the apical membrane as opposed to the basal membrane, displaying similarities with the spheroids obtained from Matrigel cultivation. Although this finding is intriguing, further studies are necessary to rationalize why the elevated lipid-ordering structure is established on the apical membrane for the DEPMiC-produced spheroids. Combined with a custom-built detection system, we also demonstrated the possibility of following drug responses from individual spheroids in an in situ manner. Looking into further validations, integration and automation of the modules of DE generation, localization, and observation are expected to promote this application in a clinical setting. Lung cancer, together with other cancers, has been a lingering public burden for human health due to the notorious heterogeneity of tumors against therapeutic treatments. The presented DEPMiC platform is expected to serve as a scalable factory for the production of tumor spheroids and yet a programmable bioreactor for long-term cultivation, microenvironmental regulation, and drug efficacy testing. More importantly, the capability of DEPMiC to analyze individual spheroids may help to advance our understanding of tumor heterogeneity and, ultimately, devise personalized treatments.

## Materials and methods

### Fabrication of the DE generator and the emulsion trapper

The master molds of the DE generator and emulsion trapper were fabricated by the widely employed soft lithography process^49^. Briefly, the designed patterns for the DE generator and emulsion trapper were illustrated by CAD software (Autodesk, San Rafael, USA) and printed on two separate transparency masks at a resolution of 25,400 DPI (MicroCAD Photo-Mask Ltd, Shenzhen, China). Following the protocol provided by the manufacturer, the patterns were transferred onto a silicon wafer coated with SU8-2075 photoresist (MicroChem, Westborough, MA, USA). After ultraviolet (UV) exposure at the proper dosage and baking, the uncrosslinked photoresist was removed by the SU8 developer (MicroChem, Westborough, MA, USA). The designated heights for the DE generator and emulsion trapper were 70 µm and 110 µm, respectively.

The PDMS prepolymer and curing agent (Dow Corning, Midland, MI, USA) were fully mixed at a ratio of 10 : 1 and poured onto the master mold. After curing at 80 °C overnight, the PDMS was peeled off and cut into slabs. For the DE generator, holes for the fluid inlet and outlet ports were produced using a 1.0 mm Miltex biopsy punch (Integra Life Sciences, NJ, USA). The PDMS strip was subsequently bonded with a piece of cover glass coated with a semicured PDMS mixture (prepolymer : curing agent = 2 : 1). The bonded chips were then baked at 80 °C in an oven overnight to cure the PDMS interface layer. For the emulsion trapper, cured and cut PDMS slabs containing microwells were assembled onto the wells of a 24-well plate or a confocal dish. Briefly, one drop of PDMS mixture (prepolymer : curing agent = 10 : 1) was dripped onto the well or the confocal dish. The PDMS slab was then placed into the well or dish followed by a gentle press. The assembled PDMS well plates or dishes were then baked at 80 °C in an oven overnight to strengthen the bonding.

### Cell culture

Calu-3 cells (ATCC catalog number HTB-55) were seeded in a T75 flask (SPL, Gyeonggi-do, Korea) and grown in minimum essential medium (Gibco, USA) supplemented with 10% fetal bovine serum and 1% penicillin–streptomycin (Gibco, USA). The cells were maintained at 37 °C in a humidified incubator supplied with 5% CO_2_ and the culture medium was replaced every 2 days. When the confluence reached ~70–90%, the cells were collected by trypsinization and transferred to the subsequent 3D culture conditions, essentially in Matrigel and DEPMiC. The 3D culture in Matrigel (Corning, NY, USA) followed a validated protocol^50^. Calu-3 cells were resuspended in undiluted Matrigel at a concentration of 1000 cells/μL. Drops of the Calu-3-Matrigel mixture in a volume of 50–100 μL were dispensed in a 12-well plate and incubated at 37 °C for 20 min. Upon solidification of the Matrigel, culture medium was added to cover the Matrigel drops. After 6–8 days of culture, the formed spheroids were collected by incubating the Matrigel with dispase II (2 mg/mL, Gibco, USA) for 30–60 min at 37 °C.

### Pretreatment by the DEs and emulsion entrapment

The collected Calu-3 cells were resuspended in fresh culture medium blended with Ficoll PM400 (25 mg/mL, GE Healthcare, USA), Ficoll PM70 (37.5 mg/mL, GE Healthcare, USA), and sodium alginate (0.2% w/v, Sigma-Aldrich, St. Louis, MI, USA) at a final concentration of 5 million cells/mL. Considering the cell concentration in the feed solution (5 million/mL) and the diameter of the produced DE (150 µm), the average number of cells in each DE was ~8.83. According to the Poisson distribution^51^, the probability of DEs containing at least one cell is as follows:

$$P(x \geq 1)=1 -p(x=0)=1-\frac{e^{-\lambda}\lambda^{k}}{k!}=1-\frac{{\rm{e}}^{-8.83}\times 8.83^{0}}{0!}=0.99985$$

In other words, the probability of the DEs containing no cells is ~2 in 10,000. Indeed, empty DEs, DEs containing no cells, were rarely observed in our experiments. Furthermore, the number of cells in each DE ranged from 3 to 11 due to the Poisson distribution.

Prior to cell encapsulation, the DE generator was surface treated. In particular, the second crossing junction was selectively rendered hydrophilic according to previously developed procedures^31^. As shown in Fig. 1a, the cells in culture medium, fluorocarbon oil (1% w/v Pico-Surf in NOVEC7500, Sphere Fluidics, Great Abington, Cambridge, UK) and culture medium containing Pluronic F-127 (2.5% w/v, Sigma-Aldrich, St. Louis, MI, USA) were introduced to the inner aqueous inlet, oil inlet, and outer aqueous inlet, respectively. Three syringe pumps (Harvard Apparatus, Holliston, MA, USA) were used to control the volumetric flow rates of the three inlets at 8, 8, and 10 μL/min, respectively. Syringes of 1 mL (Becton, Dickinson and Company, Franklin Lakes, NJ, USA) and PTFE tubes (Cole-Parmer, Vernon Hills, IL, USA) were used as connections. The generated DEs were 150 ± 4.2 µm in diameter with a shell thickness of 11.9 ± 1.7 µm (*n* = 30).

Prior to emulsion entrapment, the emulsion trapper was immersed in BSA solution (5 mg/mL) and degassed in a vacuum chamber until no bubbles were observed on the PDMS surface. The emulsion trapper was sterilized by exposure to UV in a cell culture hood for at least 30 min. Afterwards, 5 μL of DEs collected from the DE generator were gently dispensed onto the emulsion trapper by a handheld pipette. As shown in Fig. 1a, the DEs were nicely positioned into the microwells, given the geometric fit between the well and DE. By tilting the emulsion trapper slightly, the untrapped DEs could be facilely removed from the trapper due to the nonadherent surface treated by BSA adsorption.

### Cell viability in DE culture

Cells in the DEs were incubated with calcein-AM (0.5 μg/mL, Invitrogen, Waltham, MA, USA) and PI (5 μg/mL, Sigma-Aldrich, St. Louis, MI, USA) at 37 °C for 20 min. After incubation, the remaining calcein-AM and PI were washed away. Epifluorescent images were captured with a confocal microscope (Nikon C2, Tokyo, Japan) equipped with 488 and 561 nm lasers, which served as the excitation for calcein and PI, respectively. The emissions from calcein and PI were filtered through appropriate filters (525/50 nm bandpass and 561 nm longpass). Image analysis, including quantification of the total projection area and the fluorescently positive (green and red) areas, was performed by ImageJ (National Institutes of Health, Bethesda, MD, USA). The percentage of calcein- or PI-positive area was calculated as the percentage of green or red area over the total projection area for each spheroid. Statistical analysis was conducted by Prism 8.0 (GraphPad Software, San Diego, CA, USA).

### Growth measurements of the spheroids

The spheroids were released from the DEs by incubation in the emulsion-releasing agent nonafluoro-tert-butyl alcohol (Sigma-Aldrich, St. Louis, MI, USA) for 20 min. The cytotoxicity of the emulsion-releasing agent was negligible at concentrations up to 0.5% after 20 min of incubation (Supplementary Fig. S1). The projection areas of cell aggregates were quantified by contouring the projected boundary of the cells. The circularity of the projected boundary was defined as:

Circularity = 4*πA*/*P*^2^

where the projection area (*A*) and the perimeter (*P*) of the projected boundary were quantified by ImageJ.

### Raman spectroscopy

The lipid-ordering distribution in the apical and basal membranes of the cyst structures was examined by a Raman confocal microscope (LabRAMHR Evolution, Horiba, Japan) located at Guangdong University of Technology in Guangzhou, China. The laser source introduced for the measurements was at the wavelength and power of 532 nm and 10 mW, respectively. Each selected point was illuminated by the laser for 15 s and no cell damage was observed. Data from at least three different positions on the apical or basal membranes were collected. After background subtraction, the obtained Raman spectra were smoothed by the Savitzky–Golay method^52^. The resulting spectra were further normalized by the maximum value at ~2930 cm^−1^ and fitted by seven Lorentzian peaks ascribed to the different stretching vibrations of CH_2_ and CH_3_, and/or the Fermi resonance between the asymmetric CH_2_ stretching mode^42^. Data analysis was performed by OriginPro 8.1 (Originlab, Northampton, MA, USA).

### Customized fluorescence measurement platform and drug response of the spheroids

A customized platform was constructed to measure the fluorescence signal from calcein as a reporter for the drug response from each spheroid. A diode laser (488 nm, MLL-III-488L/1 ~ 50 mW, Changchun New Industries Optoelectronics Tech. Co., Ltd, Changchun, China) was employed as the excitation source and focused through an objective (S Plan Fluor, ×40, NA = 0.6, Nikon, Tokyo, Japan). Photons emitted from calcein were directed into a dichroic mirror (GCC-414002, Daheng New Epoch Technology, Beijing, China). Signals were then split by a prism (GCC401102, Hengyang Optics, China) to a camera (DFK 37BUX273, The Imaging Source Asia Co., Ltd) and to a photomultiplier tube (PMT; PMM02, Thorlabs, Newton, NJ, USA) for bright-field observation and photon counting, respectively. Prior to the PMT, the signal was filtered through a bandpass filter (525/40, Nikon, Tokyo, Japan). A digital counter (National Instruments, Austin, TX, USA) was used to integrate the fluorescence signals in 100 ms intervals for 5 cycles with a sampling frequency of 25,600 Hz. A customized LabVIEW program (National Instruments, Austin, TX, USA) was used to control the duty cycle of laser excitation (open only within each half cycle, 10 ms) and to perform data acquisition analysis.

At 5 days post culture, the spheroids were incubated with the anticancer drug vinorelbine (MedChemExpress, Monmouth Junction, USA) at titrated concentrations (0.1, 1, 5, 10, and 20 μM). In situ analysis was conducted at 3 days post drug treatment. The viability of the cells was assessed by calcein-AM staining (0.5 μg/mL). A translational stage (Thorlabs, Newton, NJ, USA), controlled by a customized LabVIEW program, was employed to scan the spheroids in the individual wells. The measured fluorescence signals were normalized to those of the untreated group before conversion to the viability of the spheroids. Notably, the currently available platform was designed to measure the averaged fluorescent signal from individual spheroids with a spot size of ~5 µm in diameter. The acquisitions of bright-field images and fluorescent signals were conducted separately.

## Supplementary information

Supporting information
