# Supplementary material for: Double emulsion-pretreated microwell culture for the in vitro production of multicellular spheroids and their in situ analysis
Source: Microsyst Nanoeng. 2021 May 24;7:38. doi: 10.1038/s41378-021-00267-w (PMC8433470; doi:10.1038/s41378-021-00267-w)
Supplement: Supplementary file 1 — Supporting information [file 41378_2021_267_MOESM1_ESM.docx]

**Supporting Information**

**Double emulsion-pretreated microwell culture for *in vitro* production of multicellular spheroids and *in situ* analysis**

Fuyang Qu^1^, Shirui Zhao^1^, Guangyao Cheng^1^, Md. Habibur Rahman^1^, Qinru Xiao^1^, Renee Wan Yi Chan^2,3^ and Yi-Ping Ho^1,4,5,6*^

^1^Department of Biomedical Engineering, Faculty of Engineering, The Chinese University of Hong Kong, Shatin, New Territories, Hong Kong SAR, China

^2^CUHK-UMCU Joint Research Laboratory of Respiratory Virus & Immunobiology, The Chinese University of Hong Kong, Shatin, New Territories, Hong Kong SAR, China

^3^Department of Paediatrics, Faculty of Medicine, The Chinese University of Hong Kong, Shatin, New Territories, Hong Kong SAR, China

^4^Hong Kong Branch of CAS Center for Excellence in Animal Evolution and Genetics, The Chinese University of Hong Kong, Shatin, New Territories, Hong Kong SAR, China

^5^The Ministry of Education Key Laboratory of Regeneration Medicine, Shatin, New Territories, Hong Kong SAR, China

^6^Centre for Novel Biomaterials, The Chinese University of Hong Kong, Shatin, New Territories, Hong Kong SAR, China

*Correspondence should be addressed to Y.-P. Ho (ypho@cuhk.edu.hk)

**MTT assay**

The cytotoxicity of DE releasing agent nonafluoro-tert-butyl alcohol was assessed by the MTT assay. Briefly, 20,000 Calu-3 cells were seeded in a 96-well plate (SPL, Gyeonggi-do, Korea). After cultured in a cell incubator (37^o^C, 5% CO_2_) overnight, the Calu-3 cells were exposed to the nonafluoro-tert-butyl alcohol (Sigma-Aldrich, St. Louis, Missouri, USA) at titrated concentrations of 0.1%, 0.5% and 1.0% (v/v) for 5, 10 and 20 min. After the treatment, cells were incubated with the MTT solution (0.5 mg/ml in the cell culture medium, Sigma-Aldrich, St. Louis, Missouri, USA) at 37^o^C for 4 hours. Subsequently, the MTT solution was removed and the resulting formazan product was dissolved in DMSO (100 μL for each well). The optical absorbance was examined at 490 nm by a microplate reader (SpectraMax M3, Molecular Devices, San Jose, CA, USA).

**Immunostaining**

Calu-3 spheroids cultured by both the Matrigel and DEPMiC were fixed in paraformaldehyde (4% w/v, Sigma-Aldrich, St. Louis, Missouri, USA) for 20 min at room temperature. After washing with PBS for three times (5 min each), the fixed spheroids were then permeabilized with Triton X-100 (0.5% v/v, Sigma-Aldrich, St. Louis, Missouri, USA) for 15 min at room temperature. Followed by an additional washing, samples were blocked by BSA (3% w/v in PBS, Sigma-Aldrich, St. Louis, Missouri, USA) for 2 hours at room temperature. Subsequently, the spheroids were incubated with rabbit polyclonal anti-ZO-1 (1:50 dilution, Cell Signalling Technology, Danvers, Massachusetts, USA) and mouse monoclonal anti-integrin β1 (1:50 dilution, Abcam, Cambridge, UK) overnight at 4oC. After washing with BSA solution (3% w/v in PBS) thoroughly, fluorescently-labelled antibodies, Alexa647-conjugated Donkey Anti-Rabbit IgG (1:200 dilution, Abcam, Cambridge, UK) and TRITC-conjugated donkey anti-mouse IgG (1:200 dilution, Abcam, Cambridge, UK), were added and incubated with the samples at 37^o^C for 1 hour. After another wash, DAPI (1 μg/mL, Abcam, Cambridge, UK) was introduced to stain the nuclei at 37^o^C for 1 hour. Followed by the final wash, the samples were then inspected by a confocal microscope (Leica TCS SP8, Leica, Wetzlar, Germany).


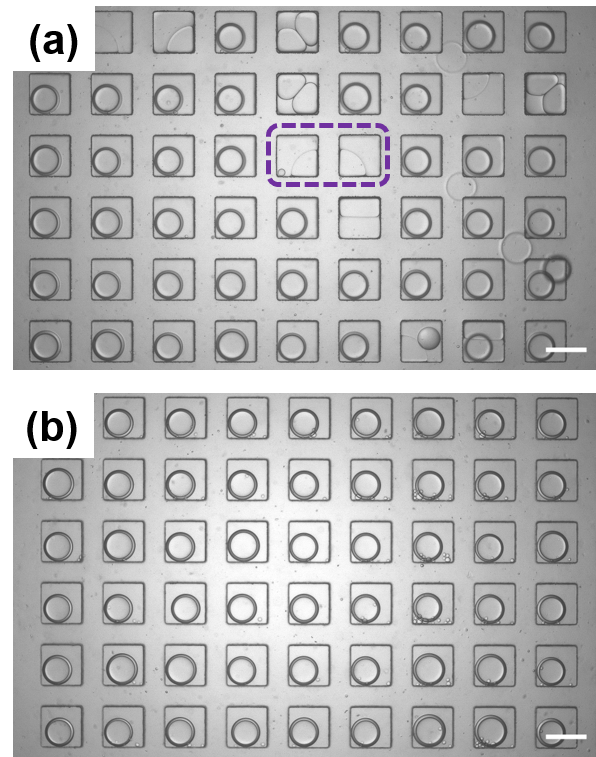


**Figure S1.** Double emulsions trapped in the microwells of emulsion trapper. (a) Double emulsions were easily broken (boxed in purple) when the surface of microwells was not treated with BSA. (b) With surface treatment of BSA, all the double emulsions remained intact in the microwells. Scale bar: 200 μm.





**Figure S2.** Cytotoxicity of nonafluoro-tert-butyl alcohol, the emulsion releasing agent employed in this study. Calu-3 cells were incubated with titrated concentrations of nonafluoro-tert-butyl alcohol, namely 0.1%, 0.5% and 1.0% (v/v), for 5, 10 and 20 min. The viability was evaluated by MTT assay.


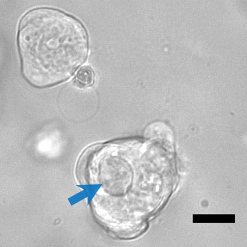


**Figure S3**. Calu-3 spheroids cultured in Matrigel for one day. The hollow structure, presumably the vacuolar apical compartments, was observed as indicated by the blue arrow. Scale bar: 20 μm.


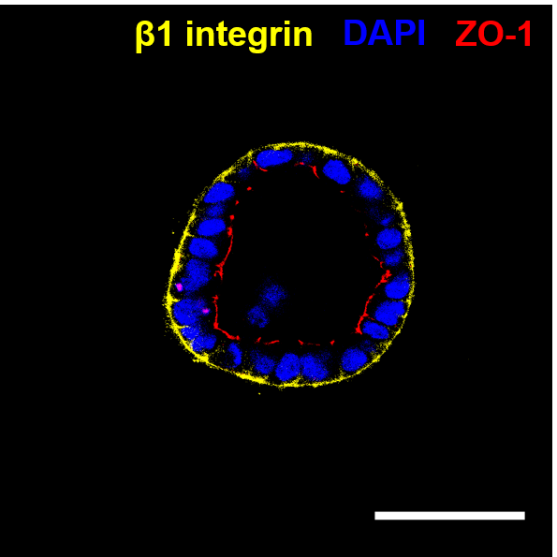


**Figure S4.** Immunostaining of the spheroid cultured in Matrigel. Shown here was the identical Calu-3 spheroid presented in **Figure 5a**. The fluorescent signals collected from β1 integrin, ZO-1 and nucleus (DAPI) were pseudocoloured as indicated accordingly. Scale bar: 50 μm.


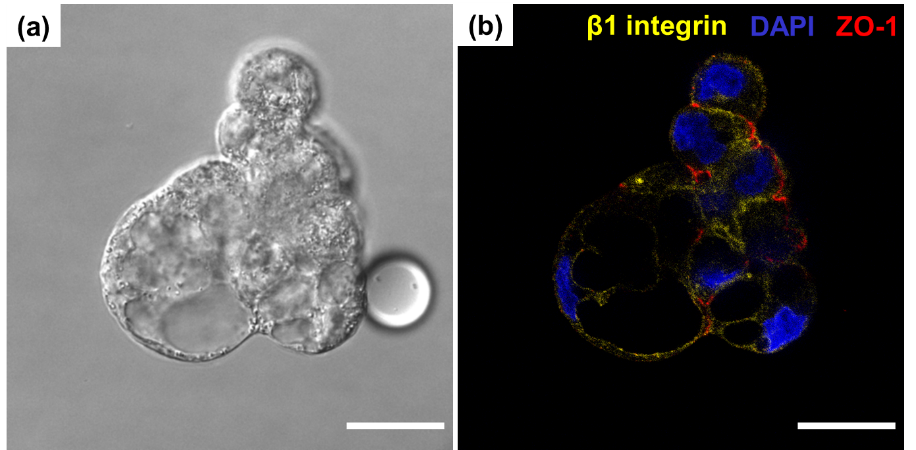


**Figure S5.** Immunostaining of one spheroid cultured by DEPMiC. (a) The bright field image and (b) Immunostaining of the spheroid. The fluorescent signals collected from β1 integrin, ZO-1 and nucleus (DAPI) were pseudocoloured as indicated accordingly. Scale bar: 20 μm.
